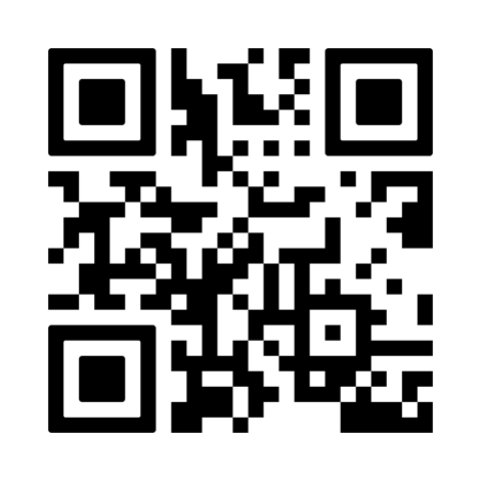# Laparoscopic morphological aspects and tentative explanation of the aetiopathogenesis of isolated endometriosis of the sciatic nerve: a review based on 267 patients 

**DOI:** 10.52054/FVVO.13.4.047

**Published:** 2021-12-30

**Authors:** M Possover

**Affiliations:** Possover International Medical Center - Zürich – Switzerland

**Keywords:** Endometriosis, sciatic nerve, pathogenesis, progenitor cells, laparoscopy

## Abstract

**Background:**

Endometriosis of the sciatic nerve (ESN) is considered a rare disease. How can endometriosis develop within the sciatic nerve; a structure which has nothing in common with the uterus either anatomically or functionally, and why it occurs in the absence of any retroperitoneal/parametric endometriosis, is unknown. A better understanding of the pathophysiology of this enigmatic disease may improve its diagnosis and therapy.

**Materials and Methods:**

From a pool of 452 patients operated for ESN, only patients with “isolated” endometriosis of the sciatic nerve” confirmed at laparoscopy were included in this study. Patients with suspicion of ESN by extension from a parametric, ovarian or other intraperitoneal deeply infiltrating endometriosis were excluded from this study.

**Main outcome measure:**

All information acquired during the preoperative patient’s medical history and clinical examination were collected and compared with the morphological aspects of the disease observed by the laparoscopic treatment. Patients were classified into three groups according to the time interval between the onset of sciatic pain and the time of surgery: less than 1 year (Group 1), between 1 and 3 years (Group 2), and more than 3 years (Group 3).

**Results:**

Two hundred sixty-seven consecutive patients were included in this study. In Group 1 (n=67), 76% of the patients presented with cyclical sciatica, without sensory or motor disorders of the lower limbs. Laparoscopic exploration found in the great majority of these patients only the presence of an isolated endometrioma in the nerve itself, the size of which was proportional to the time elapsed since the onset of pain. In Group 2 (n=83), pain had become constant in 91% of the patients with neurological disorders of the lower limb (foot drop, Trendelenburg gait, atrophied muscles) in about 30% of patients. Laparoscopic examination revealed, in addition to intraneural cystic lesions, a retroperitoneal fibrosis in more than 80% of the patients. In the third group (N=117), more than 80% of the patients presented with neurological disorders of the lower limb, with, on laparoscopic examination, massive retroperitoneal fibrosis with endometriomas in the nerve and adjacent pelvic wall muscles in all patients and an infiltration of the obturator nerve in 41% of patients.

**Conclusions:**

The different morphologic aspects of ESN do not correspond to different forms of the disease, but obviously to one single disease at different stages of its evolution. ENS starts first with the development of an endometrioma within the sciatic nerve, then develops in a second step a perineural fibrosis that expands into the whole retroperitoneal space and finally involves surrounding anatomical structures. The ESN is a very particular pathology because it induces a completely new aspect on the pathogenesis of endometriosis: all hypothesis of implanted endometrial cells following retrograde menstruation, angiogenic spread, lymphogenic spread or the metaplasia theory cannot explain the pathogenesis of this disease. ESN obviously does not develop from “genital metastatic cells”. A possible hypothesis for explanation the pathogenesis of ESN, could consist in the development of endometriosis of the nerve from progenitor stem cells present within the nerve, pluripotent cells which, for an as yet unknown reason (possibly in connection with iterative inflammations and micro-damages of the nerve itself), mutate and proliferate to form endometriosis.

## Introduction

Endometriosis is an enigmatic disease, but endometriosis of the sciatic nerve (ESN) is even more so.

Cyclical sciatic pain in women suggests potential endometriosis of the sciatic nerve and especially when there is associated difficulty in walking with resultant foot drop, which usually appears two years after the onset of pain. Absence of neurological deficits does not exclude ESN, but improvement of pain during the night may be indicative of a possible vascular nerve entrapment. It is at this diagnostic level that the subtleties of diagnosis in neuropelveology find their importance. Endometriosis of the sacral roots by deep infiltrating parametric endometriosis is relatively easy to understand, but ESN and in particular isolated forms of the disease (Possover and Chiantera, 2007) is much less so. What do endometriosis cells look for in such an isolated region of the body whose anatomical area is far removed from, and without any communication with, the peritoneal cavity and the genital organs? Surgery for ESN reveals, as in genital endometriosis, multiple aspects of the disease with the presence of haemorrhagic lesions even within the nerve itself (intraneural endometrioma) and in adjacent muscles (piriformis muscle, internal obturator muscle, psoas muscle), as well as retroperitoneal fibrosis with possible compression of adjacent nerves (obturator nerve, gluteal nerve, pudendal nerve). In order to analyse and better understand all these different aspects of this special form of non-genital endometriosis, we have reviewed all of our patients and corresponding laparoscopic procedures performed over the last 16 years for the treatment of ESN.

## Material and methods

All consecutive 452 patients who underwent laparoscopic surgery for endometriosis of the sciatic nerve (with histological confirmation) by MP since 2004 were documented in an Excel database (Microsoft Corporation, Redmond, Washington). Patients with deeply infiltrating parametric endometriosis with involvement of the sacral plexus/sciatic nerve or even extension to the sciatic nerve were excluded from this study. Only patient with ESN without any further manifestation within the peritoneal cavity and without any deep invasive endometriosis (DIE) of the rectovaginal space or of the parametries were included in this study.

In the review of the anamnestic data collected at preoperative neuropelveologic workup (Possover and Forman, 2014; Possover, 2015), particular attention was paid to the timing of the onset of pain symptoms and functional disorders for lumbosacral radiculopathy:

- Sciatic, pudendal and back pain
- Motor neurological disorders such as foot drop, difficulties for dorsal/plantar flexion of the toes/ankle (difficulty climbing stairs), reduction of Achilles reflex, Trendelenburg gait (damage of the superior gluteal nerve)
- Calf and gluteal muscle atrophy
- Sensory disorders, especially numbness in the sacral dermatomes.

Preoperative data were correlated with intraoperative findings collected during all laparoscopic procedures. For this purpose, the stored videos of all interventions were retrospectively examined for the exact localisation and aspect of the findings.

All patients signed informed consent forms prior to surgery, and provided written informed consent for the use and publication of case details, personal information, images, and videos.

## Results

Two-hundred-sixty-seven (267) consecutive patients were included in this study. The mean age of the patients was 29 years (range, 24-42). We have published previously about postoperative evolution of pain and recovery of functional disorders after such laparoscopic treatments (Possover et al., 2011; Possover, 2017). Patients were classified into three different groups, according to the time interval between the onset of sciatic pain and the time of surgery: less than 1 year, between 1 and 3 years and more than 3 years. Finally, we correlated each preoperative patient’s history and their neuropelveologic workup with the morphological aspect of the endometriosis discovered during their operation.

### Group 1: patients with less than one year between appearance of sciatic pain and surgery

**Figure 1:**
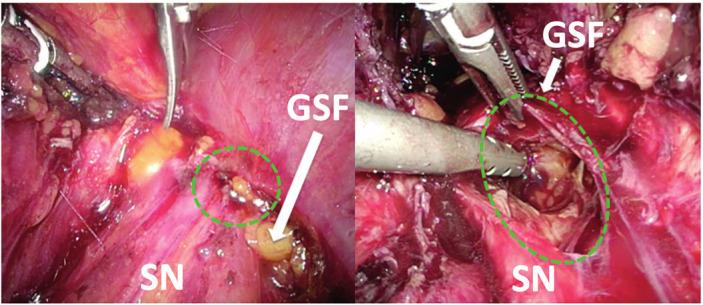
Two different samples of isolated endometriosis of sciatic nerve on the right SN; SN: sciatic nerve - GSF: greater sciatic foramen. Left: minimal lesion <2mm of the distal endopelvic portion of the SN at the level of the greater sciatic foramen (appearance of sciatic pain/surgery <6months – the patient belongs to group 1). Right: large endometrioma involving >50%endopelvic portion of the SN (appearance of sciatic pain/surgery 19 months – the patient belongs to group 2).

Sixty-seven patients were included in this group. Eleven patients presented some L5-S1 hypoesthesia but no full numbness and three some weakness of the plantar/dorsal flexion of the ankle and one a minimal Trendelenburg gait. In all other patients, no sensor-motor dysfunctions were found. Thirty-six patients had an isolated endometrioma of the sciatic nerve (without identification of any further manifestation of endometriosis in the entire pelvic cavity) (Figure 1). Surgical excision of endometrioma required an interfascicular neurolysis of the sciatic nerve for exposure of the disease. A further thirty-one patients had minor peritoneal endometriosis, in 6 patients an ovarian endometrioma and in thirteen minimal DIE of the proximal sacrouterine ligaments, but none presented any retroperitoneal DIE or a DIE of the rectovaginal septum.

All patients presented with sciatic pain, mainly cyclical when the intraneural endometrioma was less than 5mm in diameter, and constant sciatica in larger cystic lesions. In no patients of this group did laparoscopic exploration find either a massive retroperitoneal/parametric fibrosis (just perineural) or endometrioma/fibrosis of pelvic sidewall muscles adjacent to the sciatic nerve (psoas muscle, piriformis muscle or the internal obturator muscle). Neurological disorders were found only in patients with large intraneural endometrioma.

### Group 2: one to three years between appearance of sciatic pain and surgery

Eighty-three patients were classified in this group. Pain became constant in 91% of the patients with neurological disorders of the lower limb (foot drop, Trendelenburg gait, atrophied muscles) in about 30% of patients. Laparoscopic examination revealed, in addition to intraneural cystic lesions, retroperitoneal fibrosis in more than 80% of the patients and an infiltration of adjacent pelvic side muscles in 74%.

### Group 3: more than three years between appearance of sciatic pain and surgery

One hundred seventeen patients were classified in this group. In addition to sciatic pain, 41% of patients presented with neuropathic pain of the obturator nerve and a little less than 14% with homolateral pudendal pain. The vast majority of patients in this group had neurological disorders, with nearly 84% of patients experiencing foot drop. 30% presented with a Trendelenburg gait due to superior gluteal nerve damages. On laparoscopic examination, all the patients had a very intense and extensive retroperitoneal fibrosis next to the nerve lesion, with the presence of endometriosis of the adjacent muscles of the lateral pelvic area.

Laparoscopic macroscopic complete resection of endoneurial endometriosis with preservation of some continuity (at least 20%) of the sciatic nerve could be obtained in all patients in this series. (Video: https://www.youtube.com/watch?v=3_mHyXB72M8).

Results are summarised in Table I.

**Table I:** Summary of the data. Patients were classified into three groups according to the time interval between the onset of sciatic pain and the time of surgery: less than 1 year (Group 1), between 1 and 3 years (Group 2), and more than 3 years (Group 3).

	Pain Symptoms	Neurologic disorders	Intraoperative findings	
Group 1 (n=67)	Cyclical sciatica (76.12%)	Foot drop (4.47%)	Endometrioma	
	Constant sciatica (23.88%)	Trendelenburg gait (1.49%)		- intraneural (100%)
	Obturator pain (0%)	Calf atrophy (0%)		- adjacent muscles (0%)
	Pudendal pain (0%)	Gluteal atrophy (1.49%)	Retroperitoneal fibrosis 0%	
		Sacral hypoesthesia (16.4%)	Involvement obturator nerve 0%	
			Ureter stenosis 0%	
Group 2 (n=83)	Cyclical pain (8.44%)	Foot drop (33.73%)	Endometrioma	
	Constant sciatica (91.56%)	Trendelenburg gait (25.30%)		- intraneural (100%)
	Obturator pain (25.3%)	Calf atrophy (27.71%)		- adjacent muscles (74.69%)
	Pudendal pain (2.4%)	Gluteal atrophy (19.27%)	Retroperitoneal fibrosis (83.13%)	
		Sacral hypoesthesia (0%)	Involvement obturator nerve (12%)	
			Ureter stenosis (10.83%)	
Group 3 (n=117)	Constant sciatica (100%)	Foot drop (83.76%)	Endometrioma	
	Obturator pain (41%)	Trendelenburg gait (30.76%)		- intraneural (100%)
	Pudendal pain (13.67%)	Calf atrophy (68.37%)		- adjacent muscles (100%)
		Gluteal atrophy (30.76%)	Retroperitoneal fibrosis (100%)	
		Sacral hypoesthesia (100%)	Involvement obturator nerve (41%)	
			Ureter stenosis (27.35%)	

## Discussion

**Figure 2:**
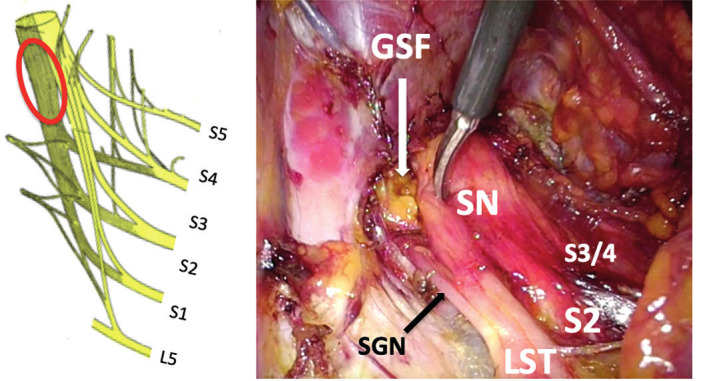
Left: Location of isolated endometriosis of the sciatic nerve. Right: exact main location (scissor tip), after emergence of the SGN, and before its exit from the pelvic cavity through the greater sciatic foramen. SN: sciatic nerve - SGN: superior gluteal nerve - GSF: greater sciatic foramen - LST: lumbosacral trunk - S: sacral nerve – L: lumbar nerve.

Endometriosis is a heterogeneous disease with a variable appearance, but in view of our data, the different morphologic aspects of ESN do not correspond to different forms of the disease, but clearly to one single disease at different stages of its evolution. According to our findings, ESN begins with the development of isolated endometriomas within the sciatic nerve (cyclic sciatic pain without neurological disorders) between the fusion of the sacral roots, after emergence of the superior gluteal nerve and before its entry through the greater sciatic foramen (Figure 2). This increases in volume due to iterative bleeding, and then secondarily develops a perineural fibrosis that extends to the rest of the retroperitoneal space, with possible involvement of the obturator nerve and of the ureter. In the advanced form of the disease, endometriomas extend to adjacent muscles (especially the internal obturator muscle) or even to the sacrospinous ligament with possible involvement of the pudendal nerve. During this evolution, the disease leads to neurogenic damage to the sciatic nerve with corresponding sensomotoric disorders. Contrary to classical beliefs that pelvic DIE progresses slowly, ESN seems to be a rapidly progressing and aggressive disease, with the onset of nerve destruction with neurological disorders already apparent between 1 and 3 years later. Because of this rapid and destructive evolution of ESN, it is necessary to evoke this disease as soon as possible. The search for sciatica must be part of the survey of any patient suffering from severe pelvic pain. The cyclical nature of the pain is symptomatic of the disease only at the beginning of its evolution. The appearance of sensory and motor disturbances of the lower limb, in particular with difficulty in climbing stairs (foot drop), walking disturbances (Trendelenburg gait) as well as disturbances of the plantar flexion of the toes are very important clinical signs.

The main question still remains of how endometriosis can develop within the sciatic nerve, and why at that special location, the distal/cranial portion of the SN before it leaves the pelvis through the greater sciatic notch. In all forms of DIE, infiltration of pelvic organs occurs per continuitam from the rectovaginal septum or form the parametries or by vascular/lymphatic metastases. Unlike endometriosis of the sacral roots which usually develops from parametric or rectal infiltration, ESN apparently develops within the sciatic nerve itself. Therefore, neither the Sampson hypothesis of implanted endometrial cells, even of stem/human endometrial epithelial progenitor cells (Cousins et al., 2017) following retrograde menstruation, nor angiogenic spread, lymphogenic spread (Mechsner et al., 2008) or the metaplasia theory can explain the pathogenesis of ESN. Also the mesoblaste theory cannot explain this form of the disease because the peripheral nervous system originates from the ectoderm via the neural crest, not from the mesoderm (Sarnat and Flores-Sarnat, 2005).

The most likely explanation could be the development of endometriosis from undifferentiated cells initially located in the nerve itself. Most researchers believe that in the peripheral nervous system, neurogenesis is only active during prenatal development, with the exception of the olfactory neuroepithelium. However, sensory ganglia in the adult peripheral nervous system have been reported to contain precursor cells that can proliferate in vitro and be induced to differentiate into neurons. The occurrence of insult-induced neurogenesis, which has been reported by several investigators of the brain, is limited to a few recent reports for the peripheral nervous system. These reports suggest that damage to the adult nervous system induces mechanisms similar to those that control the generation of new neurons during prenatal development (Czaia et al., 2012). More recently, some evidence has been reported that injured peripheral nerves provide a reservoir of mesenchymal precursor cells, which have the potential to differentiate into non- nerve lineages (Carr et al., 2019). Hidmark et al. (2017). have recently identified a population of cells within the human peripheral nerves that can respond to BMP-2 exposure or physical injury to rapidly proliferate. These cells exhibited embryonic differentiation potentials that could be induced into endothelial cells in vitro. It is proposed that these cells are possibly at the core of a previously unknown natural mechanism for healing injury (Heggeness et al., 2017). There is also evidence of spontaneous migration of endogenous or intravascular bone marrow stem cells to an injured sciatic nerve (Usach et al., 2011). The homing and engraftment of such stem/progenitor cells are influenced by injury and inflammation, presumably through the generation of a signal emanating from the damaged tissue. In turn, it is well known that bone marrow derived cells contribute to the endometrium and to endometriosis (Du and Taylor, 2007). Schwann cells, fibroblasts and macrophages also play important roles in regulating the regeneration microenvironment and improving the regeneration effect through a variety of manual interventions (Qian et al., 2019). Therefore, in peripheral nerves, stem/progenitor cells present potentials in the treatment, cure, and repair of damaged tissues secondary to injury and inflammation (Mazzeo and Santos, 2018). In such a context of inflammation and nerve injury, the sciatic nerve is especially exposed because of its very special anatomical feature: it is the largest peripheral nerve in the human body, but also the most exposed to forces of torsion, traction and pressure during daily activities (Sladjana et al, 2008). As it passes from the small pelvis to the buttocks, it changes direction, which increases the mechanical forces exerted on it all the more. The endopelvic part of the SN - where endometriosis apparently has a predilection to develop - is particularly exposed to mechanical frictional and pressure forces, and at its passage along the ilium (in the rest of its journey it is not in contact with bone) and then during its passage through the greater sciatic foramen. Such iterative mechanical frictions may potentially induce some inflammatory processes and micro-lesions, which in turn could lead to such a process of cell repair/ regeneration at that particular location in the SN.

Why such stem cells may develop endometriosis cells is unknown, but the genetic/epigenetic theory permits us to explain and understand the observation of this enigmatic disease called ESN (Koninckx et al., 2019). The set of genetic and epigenetic incidents transmitted at birth could explain the changes in a cell, which may change into stem cells and may explain why endometriosis occasionally occurs in women without an endometrium (Kawano et al., 2019; Gruenwald, 1942).

A third particularity is that all sympathetic fibres designated to the lower limb are confined to the endopelvic portion of the sciatic nerve. The sympathetic fibres of the lower limb emerge from the pelvic sympathetic trunk and anastomose at the various sacral roots, gathering at the sciatic nerve. As soon as the sciatic nerve exits the pelvic cavity, the sciatic nerve gives rise to nerve collaterals and the sympathetic fibres also spread out over different distal nerves destined for the lower limb. In rectovaginal endometriosis nodules, it has been demonstrated that there is a close morphologic relationship between these pelvic sympathetic nerves and the endometriosis foci by means of perineural and endoneurial invasion (Anaf , 2000). One particularity of the pelvic autonomous nerve system in women is that when several subpopulations of sympathetic nerves are present in the entire pelvis, only one subtype is present selectively and in large amounts in the female genital tract, the neuropeptide Y (NPY) (Possover et al., 2009). NPY sympathetic nerves are especially located in the female pelvis because of their essential role in the development of uterine neoangiogenesis in the fetus-containing uterine horn (Zukowska et al., 2002). NPY sympathetic nerves are also the most abundant in the heart and the brain (Zukowska- Grojec and Wahlestedt, 1993). If NPY has been demonstrated in SN, after sciatic nerve damages, NPY immunoreactivity appears in the SN (Ohara et al., 1994). It could be conceivable that NPY sympathetic nerves play a role in the development of ESN, but our explanation remains only a hypothesis yet to be proven.

Elucidating the mechanisms and pathways involved in the development of isolated ESN will hopefully permit the development of more specific means of prevention and therapy of this unusual and ravaging disease.

## Video scan (read QR)

https://www.youtube.com/watch?v=3_mHyXB72M8

**Figure:**